# A Case of Concurrent MPO-/PR3-Negative ANCA-Associated Glomerulonephritis and Membranous Glomerulopathy

**DOI:** 10.1155/2015/316863

**Published:** 2015-01-08

**Authors:** Yasuyuki Nakada, Nobuo Tsuboi, Yasuto Takahashi, Hiraku Yoshida, Yoriko Hara, Hideo Okonogi, Tetsuya Kawamura, Yoshihiro Arimura, Takashi Yokoo

**Affiliations:** ^1^Division of Nephrology and Hypertension, Department of Internal Medicine, The Jikei University School of Medicine, 3-25-8 Nishi-Shinbashi, Minato-ku, Tokyo 105-8461, Japan; ^2^Department of Internal Medicine, Kyorin University School of Medicine, Tokyo, Japan

## Abstract

We report a case in which antineutrophil cytoplasmic antibody- (ANCA-) associated glomerulonephritis and membranous glomerulopathy (MGN) were detected concurrently. The patient showed rapidly progressive renal deterioration. A renal biopsy showed crescentic glomerulonephritis, together with marked thickening and spike and bubbling formations in the glomerular basement membranes. Indirect immunofluorescence examination of the patient's neutrophils showed a perinuclear pattern. Enzyme-linked immunosorbent assays revealed that the ANCA in this case did not target myeloperoxidase (MPO) or proteinase 3 (PR3) but bactericidal-/permeability-increasing protein, elastase, and lysosome. The relationship between these two etiologically distinct entities, MPO-/PR3-negative ANCA-associated glomerulonephritis and MGN, remains unclear.

## 1. Introduction

The presence of antineutrophil cytoplasmic antibody (ANCA) in serum may be associated with small-vessel vasculitis, which occurs often in the renal glomeruli. Immunoglobulin deposits are usually absent in the glomeruli of patients with ANCA-associated glomerulonephritis (ANCA-GN), and ANCA infusion does not lead to glomerulonephritis in animal models. Based on these findings, it is proposed that ANCA does not damage the glomerulus directly, but neutrophils activated by ANCA integrate into capillary walls and release several protein-degrading enzymes, and, finally, these pathological changes may cause necrosis to glomerular capillary walls [1].

The two major antigens for ANCA, proteinase 3 (PR3) and myeloperoxidase (MPO), are usually referred to as the serological markers of ANCA-associated vasculitis and glomerulonephritis on ELISA tests, with perinuclear and cytoplasmic lesions in neutrophils, respectively. In these diseases, it is well-known that pauci-immune necrotizing and/or crescentic glomerulonephritis are often found in renal biopsies, with nonnephrotic range proteinuria and relatively high degrees of hematuria, as well as rapid decreases in kidney function, leading to end-stage renal disease (ESRD) within several months.

In the absence of these two major antigens for ANCA, possibilities remain for minor antigens, including elastase, bactericidal-/permeability-increasing protein (BPI), and cathepsin C. Such minor antigens often indicate drug-induced ANCA. The most common ANCA-inducing drugs are antithyroid drugs (especially propylthiouracil), though it often occurs after many years of exposure [2].

Membranous glomerulopathy (MGN) is the most common cause of nephrotic syndrome in adults. It is characterized histopathologically by subepithelial deposits of immunoglobulins and complement, with microscopic changes in the glomerular basement membrane (GBM), including spike and bubbling formations. Many cases of MGN are thought to represent primary disease, while the rest represent secondary illnesses, related to systemic lupus erythematosus, drugs, malignancies, or infections. The prognosis of MGN is variable, with one-third of untreated patients slowly progressing to end-stage renal disease within 10 years [3].

To our knowledge, no case of MPO- and PR3-negative ANCA-GN concurrent with MGN has been reported previously [4].

## 2. Case Report

**Figure 1:**
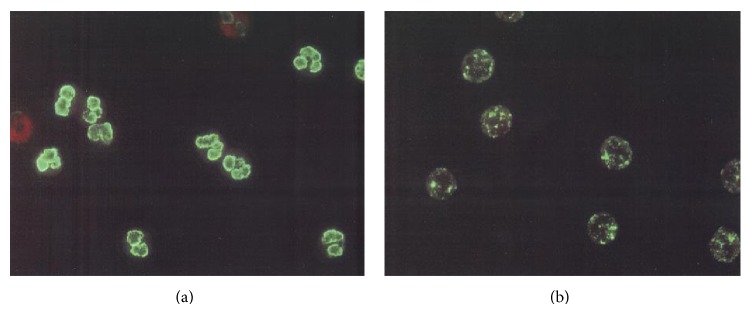
Indirect immunofluorescence reaction pattern of the patient's serum. (a) Fixed with ethanol, neutrophils showed a perinuclear pattern. (b) Fixed with formalin, neutrophils showed a cytoplasmic pattern.

The patient was a 70-year-old male with a 20-year history of sick sinus syndrome, for which he had a permanent cardiac pacemaker. He also had a 2-year history of interstitial pneumonia. While under treatment for angina pectoris 2 years before admission, he was found to have kidney dysfunction (serum creatinine, 1.4 mg/dL; blood urea nitrogen, 30 mg/dL; and 4+ protein and 2+ occult blood on urinalysis). In early December 2008, he had orthopnea, which worsened gradually. On December 24, he had a checkup in our hospital and was admitted. The medications he was taking on admission included aspirin, ticlopidine, allopurinol, carvedilol, atorvastatin, and carbocisteine. He was 171 cm tall and weighed 61 kg. His temperature was 37.0°C. His blood pressure was 145/70 mmHg. Lung auscultation revealed bilateral coarse crackles. An abdominal examination was normal. Pretibial pitting edema was evident. Laboratory findings on admission are shown in Table 1. The kidney function test had worsened, compared with 2 years earlier. There were significant hypoalbuminemia and elevation of C-reactive protein. Results of a urinalysis were 3+ positive for protein and 3+ positive for blood, with many red blood cells, 2+ for granular casts, and 1+ for red blood cell casts in the urinary sediment. The amount of proteinuria was 5.12 g/day. Urine culture results were negative on admission. An electrocardiogram showed a ventricular pacing rhythm. A chest X-ray revealed bilateral pleural effusion and pulmonary congestion. MPO and PR3-ANCA were both negative by enzyme-linked immunosorbent assay (ELISA), but P-ANCA was detected by indirect immunofluorescence (IIF; Figure 1). Bactericidal-/permeability-increasing protein (BPI), elastase, and lysozyme antibodies were also positive on ELISA (Wieslab ANCA panel kit) despite negative results for azurocidin, cathepsin G, and lactoferrin.

**Figure 2:**
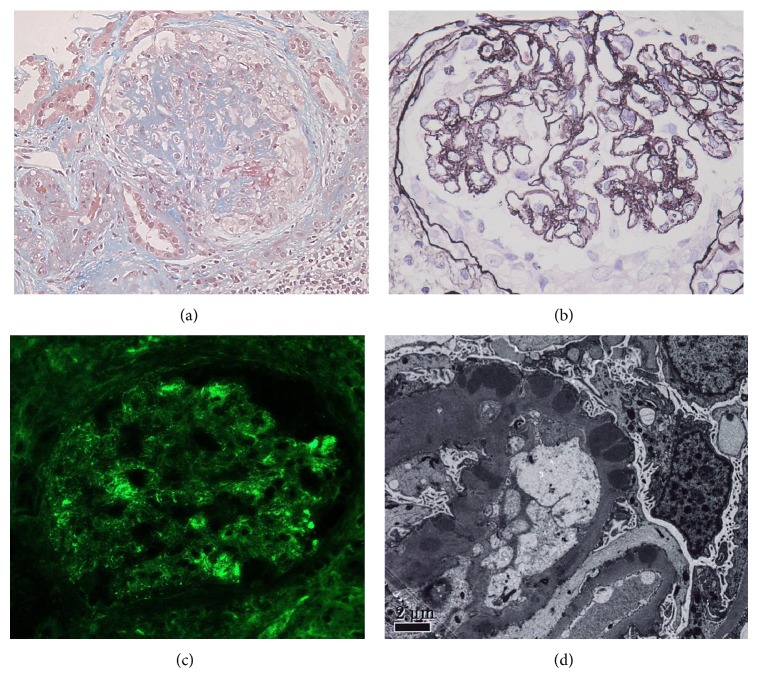
(a) Fibrocellular crescent in Bowman's space with significant collapse of glomerular tufts and the presence of glomerular-fibrinoid necrosis (Masson-trichrome stain). (b) GBM thickening including spike and bubbling formations in the subepithelial lesions (PAM stain). (c) Granular staining for IgG along glomerular capillary walls (IgG immunofluorescence stain). (d) Electron-dense deposits in the subepithelial lesions (electron microscopy).

After admission, we stopped the allopurinol and atorvastatin because several studies have shown a relationship between these drugs and the immediate development of ANCA-associated vasculitis. His pulmonary congestion was improved using diuretics. However, his kidney function worsened gradually. We performed a kidney biopsy on February 4, 2009. The renal biopsy specimen contained 15 glomeruli for light microscopic evaluation, of which 3 were globally sclerotic. There were 5 cellular, 3 fibrocellular, and 3 fibrotic crescents in the remaining 12 nonsclerotic glomeruli (Figure 2(a)). No necrotic lesion was found. Marked thickening and spike and bubbling formations were observed in the GBM by periodic acid-Schiff methenamine silver (PAM) staining (Figure 2(b)). There was tubular atrophy, especially around the globally sclerotic glomeruli, with interstitial fibrosis and inflammation involving numerous lymphocytes. An immunofluorescence microscopic evaluation revealed granular staining along glomerular capillary walls for immunoglobulin IgG (2+) and C3 (2+) (Figure 2(c)). There was no staining for IgA, IgM, or C1q. Electron-dense deposits in the subepithelial lesions and fused podocyte foot processes were revealed by electron microscopy (Figure 2(d)). Based on these findings, the present case was diagnosed histopathologically as crescentic glomerulonephritis, concurrent with MGN (Ehrenreich-Churg classification: Stage III).

Due to extraocular myositis, an inflammatory disease that selectively affects the muscles around the eyes, which occurred on January 26, 2009, he received oral prednisolone (30 mg/day) and the symptoms improved rapidly. Despite steroid therapy, his kidney dysfunction progressed severely (serum creatinine 6.23 mg/dL, urea nitrogen 54 mg/dL on February 3). After he started hemodialysis on February 12, his laboratory findings did not show significant signs of improvement in terms of kidney function. The dose of corticosteroid was tapered without recurrence of extraocular myositis.

## 3. Discussion

The pathological and physiological roles of ANCA to minor antigens, other than PR3 and MPO, have not been determined, but some cases have been reported in relation to systemic vasculitis. Our patient was positive for multiple ANCAs, including elastase, BPI, and lysozyme. Wiesner et al. reported that human neutrophil elastase antibodies (HNE-ANCA) are often found in cocaine-induced midline destructive lesions [5]. Seidowsky et al. reported three cases that developed HNE-ANCA-associated vasculitis with rapidly progressive glomerulonephritis [6]. Interestingly, both of these reports also had ANCAs for bactericidal-/permeability-increasing protein (BPI), as observed in our case. Schultz et al. reported about BPI-ANCA; the prevalence of BPI-ANCA was 5–45% in all ANCA-associated vasculitides. Other conditions with BPI-ANCA sometimes involve prolonged lower airway infection with Gram-negative bacteria [7]. Although the features of HNE-/BPI-ANCA described above were similar to our case, the typical staining pattern by IIF in HNE-/BPI-ANCA is a cytoplasmic pattern, unlike our case (perinuclear pattern).

ANCAs to multiple antigens can be seen in drug-induced ANCA-associated vasculitis, including those caused by propylthiouracil, hydralazine, penicillamine, sulfasalazine, allopurinol, and atorvastatin [2, 8, 9]. In our case, allopurinol and atorvastatin, as causal drugs of ANCA-associated vasculitis, had been prescribed many years earlier. Haroon and Devlin reported a case of ANCA-associated systemic vasculitis induced by atorvastatin but without vasculitic glomerulonephritis [8]. In addition, in the cases of atorvastatin or allopurinol, only MPO-ANCA-associated vasculitis has been reported previously. Although the possibility remains, it thus seems unlikely that the ANCA-GN observed in our case was induced by atorvastatin or allopurinol. In addition, a previous study found an association between ANCA-associated vasculitis and minor-target antigens, and the authors showed that almost 80% of cases of ANCA-associated vasculitis that were positive by immunofluorescence but negative for MPO-/PR3-ANCA by ELISA had minor targeted antigen-ANCA (BPI, elastase, cathepsin B, and lysozyme) [10]. A study on the origin and development of ANCA-associated glomerulonephritis with LAMP-2 and LAMP-2 ANCA suggested that infection and molecular mimicry may trigger autoimmunity by inducing antibodies to bacterial adhesion protein FimH and the development of AAV [11]. Indeed, the authors showed that the frequency of LAMP-2 ANCA in patients with untreated AAV was 80–91% [12]. Unfortunately, we were unable to analyze the presence of LAMP-2 ANCA in this patient because of a lack of samples.

ANCA-associated vasculitis with glomerular immune complex deposits may be associated with heavier proteinuria [13]. However, ANCA-associated glomerulonephritis rarely induces nephrotic-range proteinuria, even when immune complex deposits are demonstrated by electron microscopy (EM) in glomeruli; if present, most immune complex deposits are found in the mesangial area [13]. These observations contrast with our case, in which most of the dense deposits were found in the subepithelial area. This further supports that our case was complicated by membranous glomerulopathy.

Concurrent MGN and ANCA-associated glomerulonephritis have rarely been reported. Tse et al. reported 10 cases of MGN superimposed with vasculitic glomerulonephritis, and four of them were ANCA-positive [14]. Their kidney function recovered with immunosuppressive therapy and/or plasma exchange, except one patient in whom the renal pathological findings were especially severe. Recently, Nasr et al. reported 14 patients with MGN and ANCA-GN and identified the rate of crescent formation as a risk factor for developing ESRD [4]. However, they found that the stage of MGN was not associated with ESRD. In our case, most of the remaining glomeruli (92%) were affected by crescent formations, indicating that the kidney damage was irreversible, even with aggressive therapy.

At present, any association between MGN and ANCA-GN is unclear. Matsumoto et al. reported an interesting hypothesis that MPO demonstrated in epimembranous deposits (this lesion is anionic) is highly cationic. Because BPI, elastase, and lysozyme are all cationic, ANCAs for these minor antigens might be related to the formation of immune complexes [15]. Because of the lack of renal biopsy material, we were unable to demonstrate the presence of epimembranous deposits composed of ANCAs including BPI, elastase, and/or lysozyme, which might have revealed a correlation between ANCA-associated vasculitis and MGN in this case.

On the other hand, Nasr et al. suggested that the concurrence of MGN and ANCA-GN may just be by chance, because they occur together too infrequently to be related pathologically [4].

In our case, the presence of proteinuria, microscopic hematuria, and mild renal dysfunction two years earlier are signs of either vasculitis or more possible membranous nephropathy, which existed before the present disease. The amount of cellular crescents and the presence of interstitial inflammation suggest that vasculitis is the second disease occurring in a preexisting membranous nephropathy.

Although AAV might be associated with the development of extraocular myositis, no case of AAV with extraocular myositis has been previously reported. Thus, we also could not clarify the relationship in this case.

In conclusion, we report a rare case of MGN concurrent with ANCAGN, in which the targeted ANCA antigens were neither MPO nor PR3. Whether there is any relationship between these two etiologically distinct entities remains unclear.

## Figures and Tables

**Table 1:** Laboratory findings on admission.

Peripheral blood	
WBC	9800/*μ*L
Neutro	78.1%
RBC	311 × 10^4^/*μ*L
Hb	10.2 g/dL
Ht	31.0%
PLT	26.3 × 10^4^/*μ*L
Blood chemistry	
AST	17 IU/L
ALT	12 IU/L
LDH	286 IU/L
ALP	209 IU/L
TP	6.6 g/dL
Alb	2.7 g/dL
BUN	43 mg/dL
Cr	3.22 mg/dL
Na	144 mEq/L
K	4.3 mEq/L
Cl	111 mEq/L
Ca	8.0 mg/dL
Pi	4.0 mg/dL
TC	183 mg/dL
LDL-C	116 mg/dL
TG	121 mg/dL
FPG	83 mg/dL
HbA1c	5.5%
Serology	
CRP	5.9 mg/dL
IgG	962 mg/dL
IgA	149 mg/dL
IgM	40 mg/dL
C3	88 mg/dL
C4	19 mg/dL
CH50	30.5 U/mL
TSH	2.42 *μ*IU/mL
BNP	1135.7 pg/mL
KL-6	689 U/mL
ANA	×80 (speckled)
dsDNAIgG	<5.0 IU/mL
RA test	(−)
MPO-ANCA	<10 E.U.
PR3-ANCA	<10 E.U.
Azurocidin-ANCA	(−)
BPI-ANCA	(+)
Cathepsin G-ANCA	(−)
Elastase-ANCA	(+)
Lactoferrin-ANCA	(−)
Lysozyme-ANCA	(+)
SS-A/RO	(−)
SS-B/LA	(−)
Anti-GBM	<10 E.U.
Cryoglobulin	(−)
HBs-Ag	(−)
HCV-Ab	(−)
TPHA	(−)
Urine	
U-protein	5.13 g/day
24-hour-CCr	21 mL/min
Sediment	
RBC	Many/HPF
WBC	50–99/HPF
C-granule	2+
C-RBC	1+
